# Infarto Agudo do Miocárdio com Supradesnivelamento do Segmento ST Tratado com Intervenção Coronária Percutânea Primária: A Importância de Dados Locais

**DOI:** 10.36660/abc.20220557

**Published:** 2022-08-25

**Authors:** José C. Nicolau

**Affiliations:** 1 Hospital das Clínicas Faculdade de Medicina Universidade de São Paulo São Paulo SP Brasil Instituto do Coração (InCor), Unidade de Coronariopatia Aguda, Hospital das Clínicas, Faculdade de Medicina, Universidade de São Paulo (HCFMUSP), São Paulo, SP – Brasil

**Keywords:** Infarto Agudo do Miocárdio com Supradesnivelamento do Segmento ST, Intervenção Coronária Percutânea, Reperfusão Miocárdica, Terapia Fibrinolítica, Epidemiologia, Mortalidade

Castro et al.,^1^ devem ser elogiados por fornecerem à comunidade científica uma publicação útil que analisa os preditores de mortalidade por todas as causas em pacientes com infarto agudo do miocárdio com supradesnível do segmento ST (IAMCSST) submetidos à intervenção coronária percutânea primária (ICPP).

A ICPP é o método preferido de reperfusão para pacientes com IAMCSST que se apresentam dentro de 12 horas de evolução do início dos sintomas.^2^ Do que seja do meu conhecimento, a metanálise mais recente que comparou terapia fibrinolítica com ICPP encontrou *odds ratios* de 0,73 (p = 0,002), 0,38 (p < 0,001), 0,38 (p < 0,001) e 1,03 (p = 0,86), respectivamente para morte por todas as causas, reinfarto, acidente vascular cerebral e sangramento maior. Porém, a ausência de ampla disponibilidade de laboratórios de cateterismo e problemas logísticos, principalmente relacionados ao transporte, limitam o acesso dos pacientes com IAMCSST a essa forma de tratamento.^3^ Isso é destacado na presente publicação, onde apenas 0,26% da população analisada era da vasta região Norte do Brasil, enquanto a maioria (58%) era da região Sudeste. Talvez mais importante seja o fato de que a utilização de terapias de reperfusão (fibrinolíticos ou ICPP) em geral está longe do ideal no Brasil, e importantes diferenças regionais têm sido observadas.^4 , 5^ É importante ressaltar que a metanálise anteriormente citada de Fazel et al.,^3^ encontrou odds ratios de 0,79, 0,53, 0,70 e 1,19 para morte por todas as causas, reinfarto, acidente vascular cerebral e sangramento grave, respectivamente, ao comparar o tratamento farmacoinvasivo e a abordagem com tratamento fibrinolítico, que pode ser uma opção para problemas relacionados ao acesso à ICPP.

A comparação entre o artigo de Castro et al.^1^ e a literatura resumida na Tabela 3 deve ser interpretada com cautela. Primeiro, o artigo brasileiro analisou apenas a fase intra-hospitalar, enquanto os demais analisaram períodos de seguimento que variaram de 30 dias a 1 ano. Segundo, nem todos os estudos estavam relacionados a pacientes submetidos à ICPP. Por exemplo, o “DynTIMI”^6^ foi derivado do estudo ExTRACT, que testou o papel da enoxaparina *versus* heparina não fracionada em pacientes com IAMCSST, e o GRACE^7^ foi um registro internacional com uma ampla população de pacientes com síndromes coronárias agudas (com ou sem supradesnível do segmento ST). Vale ressaltar que todos os estudos incluídos na Tabela 3 desenvolveram escores de risco para facilitar a compreensão e facilitar a utilização dos resultados na prática diária, o que não foi o caso da publicação de Castro et al.^1^ Os autores perderam uma excelente oportunidade para desenvolver um escore de risco baseado em uma população brasileira, assim como fizeram algumas publicações anteriores do Brasil.^8^

De maneira um tanto simplificada, Castro et al.,^1^ afirmaram que o único fator de risco da Central Nacional de Intervenções Cardiovasculares (CENIC) que não estava de acordo com as demais publicações era o sexo feminino. O papel do sexo feminino como fator de risco prognóstico no IAMCSST é motivo de discussão há décadas, com alguns estudos, como o presente, concluindo que o sexo feminino é um fator de risco independente para pior prognóstico, e outros concluindo o contrário.^9^ Talvez a melhor explicação seja aquela fornecida na publicação clássica de Vacarinno et al.,^10^ há muitos anos, a saber, que existe uma interação entre sexo, idade e mortalidade por infarto do miocárdio, onde mulheres mais jovens, mas não mulheres mais velhas, apresentam maiores taxas de óbito hospitalar do que homens da mesma idade;^10^ no entanto, publicação recente sugeriu que outras interações também podem ser importantes.^11^ Em uma análise mais detalhada, podemos notar que existem muitas outras diferenças entre o estudo brasileiro e os demais. Por exemplo, o escore de risco CADILLAC incluiu 7 variáveis, incluindo anemia e insuficiência renal,^12^ e o escore ALPHA teve apenas 5 variáveis, incluindo frequência cardíaca, necessidade de suporte de vida e acesso arterial, atingindo uma estatística c impressionante de 0,88 para mortalidade por todas as causas em 30 dias.^13^

Em resumo, o estudo de Castro et al.,^1^ contribui para uma melhor compreensão da epidemiologia do IAMCSST no Brasil, com um número robusto de aproximadamente 27.000 pacientes analisados. Sua principal limitação é provavelmente um potencial viés de inclusão, uma vez que a contribuição dos pesquisadores foi espontânea e, talvez mais importante, foi perdida a oportunidade de se desenvolver e validar um escore brasileiro para pacientes com IAMCSST submetidos à ICPP.
