# Supplementary material for: Infarto Agudo do Miocárdio com Supradesnivelamento do Segmento ST Tratado com Intervenção Coronária Percutânea Primária: A Importância de Dados Locais
Source: Arq Bras Cardiol. 2022 Aug 25;119(3):458–9. [Article in Portuguese] doi: 10.36660/abc.20220557 (PMC9438528; doi:10.36660/abc.20220557)
Supplement: Translation

# Translation: ST-Elevation Acute Myocardial Infarction Treated with Primary Percutaneous Coronary Intervention: The Importance of Local Data

**Authors:** José C. Nicolau

**Affiliations:** 1 Brazil Instituto do Coração (InCor), Unidade de Coronariopatia Aguda, Hospital das Clínicas, Faculdade de Medicina, Universidade de São Paulo (HCFMUSP), São Paulo, SP – Brazil

**Keywords:** ST Elevation Myocardial Infarction, Percutaneous Coronary Intervention, Myocardial Reperfusion, Fibrinolityc Therapy, Epidemiology, Mortality

Castro et al.^1^ should be commended for providing the scientific community with a useful publication analyzing predictors of all-cause mortality in patients with ST-elevation acute myocardial infarction (STEMI) who are submitted to primary percutaneous coronary intervention (PPCI).

PPCI is the preferred method of reperfusion for patients with STEMI presenting within 12 hours of evolution from onset of symptoms.^2^ To the best of my knowledge, the most recent meta-analysis comparing fibrinolytic therapy with PPCI found odds ratios of 0.73 (p = 0.002), 0.38 (p < 0.001), 0.38 (p < 0.001), and 1.03 (p = 0.86) for all-cause death, reinfarction, stroke, and major bleeding, respectively. However, absence of widespread availability of catheterization laboratories and logistical problems, mainly related to transportation, limit the access of patients with STEMI to this form of treatment.^3^ This is highlighted in the present publication, where only 0.26% of the analyzed population was from the vast North Region of Brazil, whereas the majority (58%) was from the Southeast Region. Maybe more importantly, the utilization of reperfusion therapies (fibrinolytics or PPCI) in general is far from ideal in Brazil, and important regional differences have been observed.^4 , 5^ Importantly, the previously cited meta-analysis by Fazel et al.^3^ found odds ratios of 0.79, 0.53, 0.70, and 1.19 for all-cause deaths, reinfarction, stroke, and major bleeding, respectively, when comparing the pharmacoinvasive approach with fibrinolytic treatment, which can be an option for issues related to access to PPCI.

The comparison between the paper by Castro et al.^1^ and the literature summarized in Table 3 must be interpreted carefully. First, the Brazilian paper analyzed only the in-hospital phase, while the others analyzed follow-ups ranging from 30 days to 1 year. Second, not all studies

were related to patients submitted to PPCI. For example, the “DynTIMI”^6^ was derived from the ExTRACT study, which tested the role of enoxaparin versus non-fractioned heparin in patients with STEMI, and the GRACE^7^ was an international registry with a broad population of patients with acute coronary syndromes (with or without ST-elevation). It is noteworthy that all the studies included in Table 3 developed risk scores to facilitate understanding and to increase the utilization of the results in daily practice, which was not the case of the publication by Castro et al.^1^ The authors missed an excellent opportunity to develop a risk score based on a Brazilian population, as did some previous publications from Brazil.^8^

In a somewhat simplistic manner, Castro et al.^1^ stated that the only risk factor from the National Registry of Cardiovascular Interventions (CENIC, acronym in Portuguese) not in agreement with the other publications was female sex. The role of female sex as a prognostic risk factor in STEMI has been a matter of discussion for decades, with some studies, such as the present one, concluding that female sex is an independent risk factor for worse prognosis and others concluding that it is not.^9^ The best explanation was, perhaps, the one provided in the classical publication by Vacarinno et al.^10^ many years ago, namely, that there is an interaction between sex, age, and mortality in myocardial infarction with younger women, but not older women, having higher rates of in-hospital deaths than men of the same age;^10^ however, a more recent publication suggested that other interactions may be important as well.^11^ In a more detailed analysis, we can note that there are many other differences between the Brazilian study and the others. For example, the CADILLAC risk score included 7 variables, including anemia and renal insufficiency,^12^ and the ALPHA score had only 5 variables, including heart rate, need for life support, and arterial access, reaching an impressive c-statistic of 0.88 for all-cause mortality at 30 days.^13^

In summary, the study by Castro et al.^1^ contributes to a better understanding of the epidemiology of STEMI in Brazil, with a robust number of approximately 27,000 patients analyzed. Its main limitation is likely a potential inclusion bias, since the contribution of the investigators was spontaneous, and, maybe more importantly, an opportunity to develop and validate a Brazilian score for patients with STEMI submitted to PPCI was missed.
